# Turbine-to-Textile: Upcycling Wind Turbine Blade Waste into High-Performance PAN Composite Fibers

**DOI:** 10.1021/acsapm.5c02466

**Published:** 2025-10-17

**Authors:** Varunkumar Thippanna, Xiao Sun, M. Taylor Sobczak, Arunachalam Ramanathan, Taylor G. Theobald, Ian Doran, Joshua Were, Libin Yang, James Jaraczewski, James Casey, Vladislav V. Klepov, Liang Liang, Stephen Nolet, Arunachala Nadar Mada Kannan, Xin Xu, Kenan Song

**Affiliations:** 1 Mechanical Engineering, College of Engineering, 1355 University of Georgia, 302 E. Campus Road, Athens, Georgia 30602, United States; 2 Department of Mechanical and Industrial Engineering, College of Engineering, Northeastern University, 360 Huntington Avenue, Boston, Massachusetts 02115, United States; 3 Department of Chemistry, University of Georgia (UGA), 302 E. Campus Road, Athens, Georgia 30602, United States; 4 123DTechs., Inc., Athens, Georgia 30602, United States; 5 TPI Composites, Scottsdale, Arizona 85253, United States; 6 The Polytechnic School (TPS), Ira Fulton Schools of Engineering, Arizona State University, Mesa, Arizona 85212, United States; 8 Mechanical Engineering, School of Environmental, Civil, Agricultural and Mechanical Engineering (ECAM) & School of Chemical, Materials and Biomedical Engineering, College of Engineering, University of Georgia (UGA), 302 E. Campus Road, Athens, Georgia 30602, United States

**Keywords:** Wind turbine blades, crystallization, multilayered fibers, fiber kinetics, interfacial bonding

## Abstract

Recycling wind turbine blades (WTBs) is challenging due to their thermoset glass fiber-reinforced plastics (GFRPs), which resist chemical and thermal processing. Current methods yield low-value byproducts, underscoring the urgent need for scalable, high-value upcycling technologies. This study explores the reinforcement of polyacrylonitrile (PAN) matrix fibers using glass fibers (GFs) recovered from WTB, aiming to develop high-performance, sustainable composite materials. A systematic investigation was conducted to assess the influence of both GF concentration and layer number on the crystallinity and mechanical properties of PAN fibers. Structural evolution was characterized using differential scanning calorimetry (DSC), X-ray diffraction (XRD), and dynamic mechanical analysis (DMA), while mechanical behavior was evaluated through tensile testing. For the 256-layered fibers, the incorporation of GF increases the activation energy for cyclization by 17.75%, rising from 114.36 kJ/mol in pure PAN fibers to 134.56 kJ/mol in PAN-GF composites. XRD analysis also revealed a significant increase in crystallinity from 46.33% in PAN to 68.56% in PAN-GF. A corresponding increase in crystallite size was observed, suggesting that GF serves as a structural template, promoting PAN chain alignment and enhanced microstructural ordering. This integrated approach demonstrates the effectiveness of incorporating GF, thereby providing valuable insights into the relationship between fiber architecture and interfacial engineering while highlighting a promising pathway for upcycling end-of-life WTB components into advanced functional materials.

## Graphical abstract

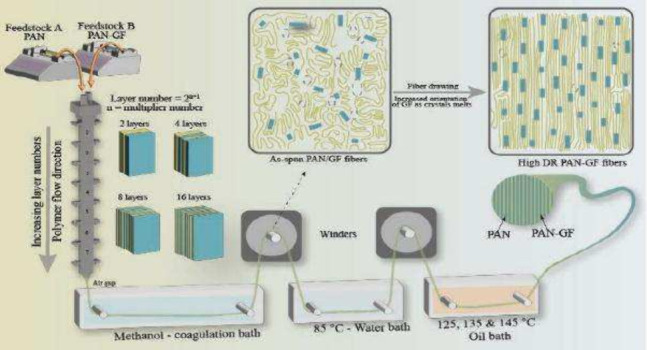

## 1 Introduction

Wind turbine blades (WTBs), primarily composed of thermoset glass fiber-reinforced plastics (GFRPs), pose a significant challenge to end-of-life recycling efforts.^1,2^ As of 2020, over 40 million tons of composite materials from WTBs are projected to accumulate globally by 2050, with the U.S. alone contributing more than 720,000 tons by 2040.^3–5^ While metals and some thermoplastics from turbines are readily recyclable, GFRP components remain largely unrecyclable due to their cross-linked polymer networks, which resist chemical depolymerization and thermal remolding.^6,7^ Current recycling methods, such as mechanical shredding, coprocessing in cement kilns, and pyrolysis, often result in low-value fillers or energy recovery, rather than material reuse.^8–10^ No efficient chemical separation or high-value upcycling technique has been widely adopted for WTB-derived GFRPs, leaving a pressing need for innovative, scalable strategies to repurpose these durable composite wastes.^11,12^

Traditional recycling of WTB composites often requires energy-intensive sorting of constituent materials, significantly driving up processing costs and limiting commercial viability.^13^ An unsorted recycling approach, wherein shredded WTB-derived composites are used directly as fillers or reinforcements without separating fibers and matrix, offers a cost-effective alternative by bypassing complex separation steps.^14^ This streamlined method not only lowers material handling and processing expenses but also enables the reuse of composite waste in structurally demanding applications.^15^ Building on this concept, the current focus shifts toward developing fiber-reinforced composites, such as those incorporating waste-derived glass or carbon fibers, that can potentially be recycled into new-generation WTBs.^16^ In particular, carbon fiber-reinforced turbine blades, which are gaining popularity due to their superior strength-to-weight ratio, may benefit from closed-loop recycling strategies where carbon-rich precursors are upcycled into high-performance composite components, advancing both sustainability and performance in the wind energy sector.^17,18^

Polyacrylonitrile (PAN) fibers play a vital role in advanced materials manufacturing, not only as key components in textiles but more importantly as the predominant precursor for high-performance carbon fibers.^19,20^ The structural integrity and mechanical performance of carbon fibers are largely dictated by the quality of the PAN precursors, which depend on factors such as molecular orientation, crystallinity, and phase uniformity.^21^ In this context, PAN fibers offer a unique advantage when reinforced with waste turbine-derived glass fibers (WT-GFs), as they can effectively encapsulate and align these fillers during spinning and stabilization.^22^ This synergy enhances the mechanical strength of the composite fibers while preparing the structure for efficient carbonization. The PAN matrix also facilitates the pyrolysis of other organic additives and polymers embedded in the waste without requiring extensive sorting of the glass fibers, simplifying processing and reducing costs.^23^ When subjected to high-temperature treatments, PAN-based composites allow selective carbonization of the polymer phase while the embedded GF remain thermally stable, enabling their continued reinforcement role in the final carbon fiber product.^24^ This makes PAN-GF composites particularly attractive for closed-loop recycling strategies, where unsorted WTB waste can be upcycled into functional carbon fiber materials for structural high-performance applications.

This study presents a sustainable strategy to enhance PAN fibers by incorporating GFs recovered from WTB materials. These recycled GFs serve not only as mechanical reinforcements but also as crystallization templates, promoting ordered polymer chain alignment during fiber spinning and drawing. The templating effect of GFs leads to increased crystallinity and larger crystallite sizes in the PAN matrix, which correlates strongly with the observed improvements in tensile strength and modulus. Through strong interfacial interactions, the GFs facilitate more efficient stress transfer and molecular orientation, resulting in enhanced mechanical performance and thermal stability. This dual role, as both structural reinforcement and nucleation agent, amplifies the functional value of waste-derived GFs in composite fiber systems. Furthermore, incorporating unsorted WTB-derived GFs into PAN fibers supports circular economy goals by transforming industrial waste into high-value, high-performance composite materials. These findings demonstrate the feasibility of developing sustainable PAN-based fibers suitable as carbon fiber precursors, while reducing environmental impact and manufacturing costs.

## 2 Results and Discussion

The multilayered dry-jet wet spinning (Figure 1a) approach enables the continuous layering of dual feedstocks (Figure 1a_1_) into complex fiber architectures. Feedstock A consists of 10 wt % PAN, while Feedstock B contains PAN with 1–4 wt % WTB waste relative to the PAN content. This processing utilizes a custom-designed spinneret containing multiple multiplying elements as the layer number follows a 2^n+1^ relationship, where the number of multipliers (n) dictates the resulting lamellar structure (Figure 1a_2_).^25^ By increasing n, the number of layers increases exponentially, leading to the creation of micro- to nanoscale layer confinement within the fibers.^26,27^ The solution passed through a methanol coagulation bath, which initiated solidification and induced molecular alignment along the fiber axis due to high shear forces (Figure 1a_3_).^28^ Following coagulation, the fibers underwent a multistage drawing process, initially in water at 85 °C, and subsequently in an oil bath at progressively higher temperatures of 125, 135, and 145 °C (Figure 1a_4_). This hierarchical structuring offers fine control over fiber morphology and interfacial composition, facilitating tailored mechanical and thermal properties (Figure 1a_5_).

**Figure 1:**
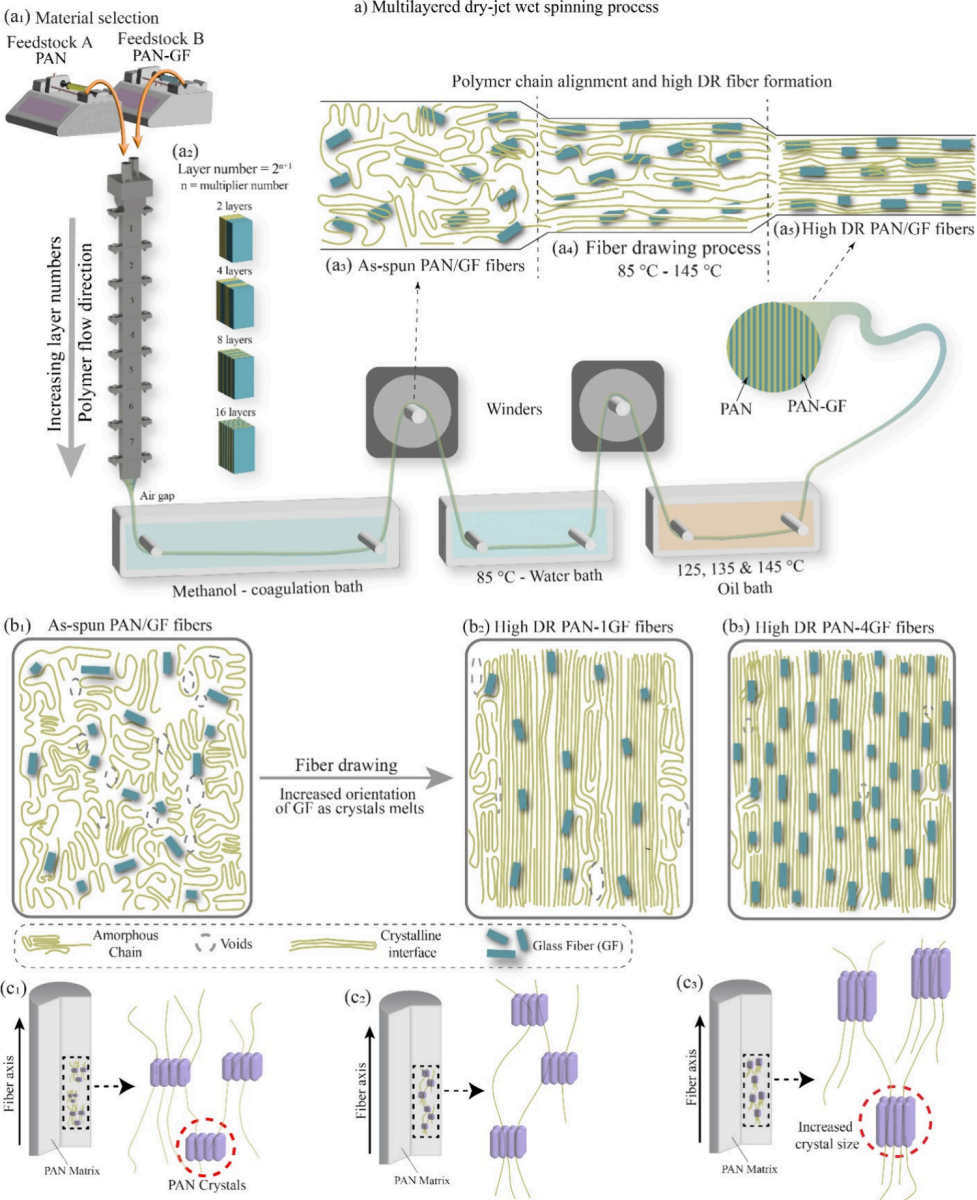
Schematic overview of the PAN-GF composite fibers with multilayered architecture and optimized properties. (a) Multilayered dry-jet wet spinning process using two feedstocks (a_1_), where increasing the number of layer multipliers (a_2_) results in as-spun fibers with complex lamellar structures (a_3_). These fibers then undergo a multistage drawing process: initially in water at 85 °C, followed by sequential oil baths at 125, 135, and 145 °C (a_4_) to achieve high draw ratios (a_5_). (b_1_–b_3_) Illustration of glass fiber (GF) alignment within the PAN matrix during postspinning drawing. (c_1_–c_3_) Fibers with higher GF content and draw ratios exhibit improved PAN chain alignment, increased crystallite size, and enhanced structural order.

During the multiplying and extrusion processes, the inclusion of WT-GFs into one of the feedstocks allows for their alignment and uniform dispersion along the fiber axis. This orientation is achieved through the shear and elongational flow fields generated within the spinneret and is further enhanced during postspinning drawing steps.^29^ The aligned GF within the PAN matrix acts not only as a physical reinforcement but also as an in situ template that drives microstructural evolution.^30^ This results in well-ordered and compact lamellar composites with superior interfacial stability (Figure 1b_1_–1b_3_).

More significantly, the embedded GF templating influences the crystallization behavior of PAN during the fiber drawing and heat treatment stages.^31^ As illustrated in Figure 1c_1_–1c_3_, fibers with higher GF content and draw ratios exhibited enhanced PAN chain alignment, increased crystallite size, and more defined crystalline interfaces. These changes reflect improved chain orientation, reduced voids, and better packing density, all of which are critical for mechanical performance.^32^ The GF served not only as a filler but also catalyzed heterogeneous crystallization, producing fibers with improved modulus and tensile strength.

### 2.1 Kinetics Analysis of Fibers

The thermal behavior of PAN and PAN/GF composite fibers during cyclization and oxidation was evaluated using differential scanning calorimetry (DSC) under both nitrogen and air atmospheres, as shown in Figure 2. In the nitrogen environment (Figure 2a_1_ for PAN and Figure 2a_2_ for 10PAN-1GF), all samples displayed exothermic peaks corresponding to the cyclization of nitrile groups into conjugated ladder-like structures, an essential transition for carbon fiber precursor stabilization.^33^ As the heating rate increased from 5 to 15 °C/min, the exothermic peaks consistently shifted to higher temperatures (Table S1), indicative of the reduced reaction time and thermal inertia effects at elevated heating rates. Notably, the PAN-GF composites exhibited higher peak temperatures and narrower exothermic transitions compared to neat PAN, suggesting improved thermal stability and more efficient molecular rearrangement.^34^ These observations were quantified using Kissinger analysis (eq 1),^35^ which revealed a significant increase in activation energy for cyclization in 10PAN-1GF compared to 10PAN (Figure 2a_3_). This trend confirms that GF fillers enhance energy requirements for initiating cyclization, potentially promoting a more ordered polymer structure and delaying premature degradation (Table S2 and Figure S1).

**Figure 2:**
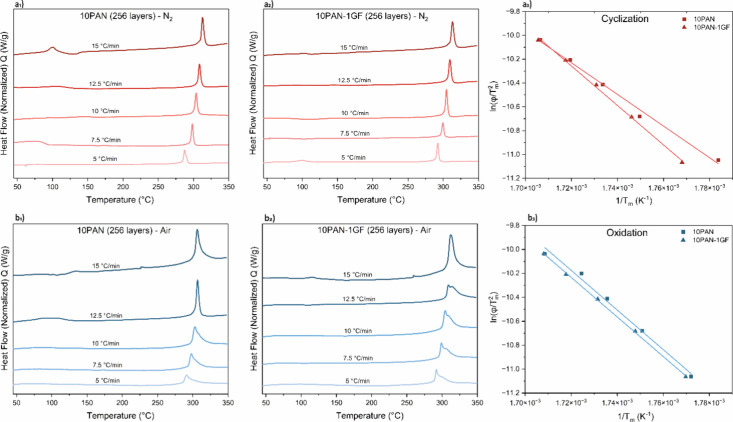
DSC curves of (a_1_) 10PAN and (a_2_) PAN-1GF fibers at different heating rates in a nitrogen atmosphere, and (b_1_) 10PAN and (b_2_) PAN-1GF fibers in an air atmosphere. Kissinger plots of ln­(ϕ/T^2^_m_) versus 1/T_m_ are shown for (a_3_) cyclization in nitrogen and (b_3_) oxidation in air, revealing the reaction kinetics of PAN and PAN/GF composite fibers. The tests were performed from room temperature (25 °C) to 350 °C at a heating rate of 5 to 15 °C/min.

In the oxidative environment (Figure 2b_1_ and 2b_2_), the DSC traces broadened, with additional shoulders and overlapping peaks emerging at lower heating rates (e.g., 5 °C/min), especially in PAN-GF composites. These features correspond to concurrent oxidation and cross-linking reactions, critical for stabilizing PAN prior to carbonization.^36^ Figure 2b_3_ presents Kissinger plots for oxidation, where 10PAN-1GF again demonstrates elevated activation energy over the neat PAN system. This implies that the addition of GF fillers delays the onset of oxidation and allows for more controlled structural rearrangement, factors that are crucial for avoiding fiber fusion and maintaining morphology during high-temperature treatment.^37^

The thermodynamic analysis using DSC also allowed quantification of enthalpy changes associated with the thermal transitions, showing consistent increases in enthalpy with higher GF concentrations and draw ratios (Table S3 and Figure S2). This supports the hypothesis that GF, acting as a reinforcement and nucleating agent, interacts strongly with the PAN matrix to promote a denser, more crystalline structure during drawing and thermal treatment. These interactions increase the thermal resistance and mechanical robustness of the fiber, evidenced by more distinct crystallization behavior and improved activation energy metrics.^38^

To explore the kinetics of thermal transitions further, the Kissinger equation was applied:

$$\frac{-\mathrm{Ea}}{R}=\frac{d\left[\ln\left(\frac{\phi }{T_{m}^{2}}\right)\right]}{d\left(\frac{1}{T_{m}}\right)}$$ 1

where Ea is the activation energy (kJ/mol), ϕ is the heating rate (°C/min), R is the molar gas constant, and *T*_m_ is the peak temperature (K), Ea was taken as the slope of the plots.

As summarized in Table 1, the composite fiber (10PAN-1GF) consistently exhibited higher activation energies, indicating a more energy-intensive but controlled reaction pathway compared to the neat PAN fibers. Several factors contribute to this behavior. The well-dispersed and partially aligned GF within the PAN matrix not only acts as a physical barrier but also serves as a crystallization template, enhancing molecular orientation during fiber drawing and providing a structural framework that stabilizes the matrix during heating.^39^ This effect becomes particularly important in the high draw-ratio (DR6) fibers used in this analysis, where extended chain alignment amplifies the influence of GF on both the thermal and mechanical performance of the precursor fibers. Additionally, the interface between the GF and PAN may induce localized stress fields or restrict polymer chain mobility, both of which can raise the energy barrier for reaction initiation. The result is a more uniform and delayed thermal response, characterized by elevated activation energies and more predictable reaction pathways. Furthermore, the reduction in voids due to fining layer formation and the increased crystallinity observed in drawn composite fibers can be attributed to the enhanced diffusion pathways and constrained chain relaxation imparted by GF fillers.^40^

**Table 1:** Activation Energies (kJ/mol) Were Determined from the Kissinger Method for 256-Layered 10PAN and 10PAN-1GF

256-layered fibers	10PAN (kJ/mol)	10PAN-1GF (kJ/mol)
Oxidation (Air)	134.87	138.43
Cyclization (N_2_)	114.27	134.56

As a result, the DSC analysis reveals that GF inclusion in PAN composite fibers significantly influences thermal behavior during stabilization. GF increases the activation energy for both cyclization and oxidation, shifts peak temperatures upward, and enhances structural order. Thus, these findings validate our hypothesis of the role of GF as a reinforcement and a processing aid, enabling the development of higher-performance PAN-based carbon fiber precursors with tailored thermal and mechanical properties.

### 2.2 Influence of Glass Fiber on PAN Crystallization

The crystallographic structure and molecular orientation of multilayered PAN and PAN/GF composite fibers were extensively investigated using X-ray diffraction (XRD) to quantify the impact of GF concentration on PAN crystallinity and crystal size. Measurements were conducted on high DR 256-layered fibers using both meridional (0° to fiber axis) and equatorial (90° to fiber axis) scan configurations to evaluate anisotropy and chain alignment as seen in Figures 3 and 4. XRD patterns revealed two dominant peaks located at approximately 2θ ≈ 17° and 29.5°, corresponding to the (110) and (020) crystallographic planes of PAN.^41^ These peaks signify a pseudohexagonal crystalline structure, characterized by two-dimensional ordering in the transverse direction.^42^

**Figure 3:**
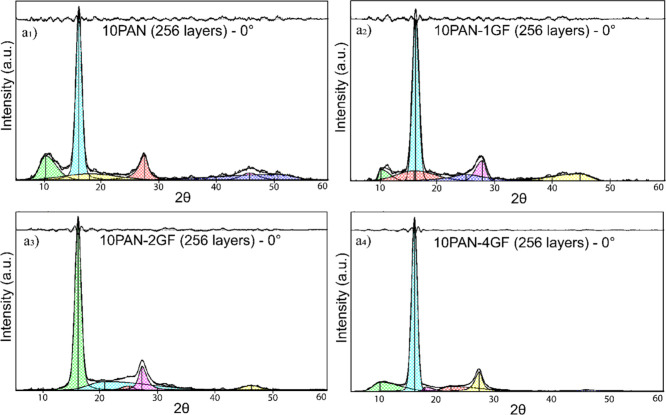
XRD pattern of PAN showing the separation of crystalline and amorphous regions to evaluate the relative degree of crystallinity. Crystallinity was determined by integrating the areas under the resolved peaks after subtracting a linear baseline established from 2θ = 5° to 60°. (a_1_–a_4_) XRD results for 256-layered fibers oriented at 0°.

**Figure 4:**
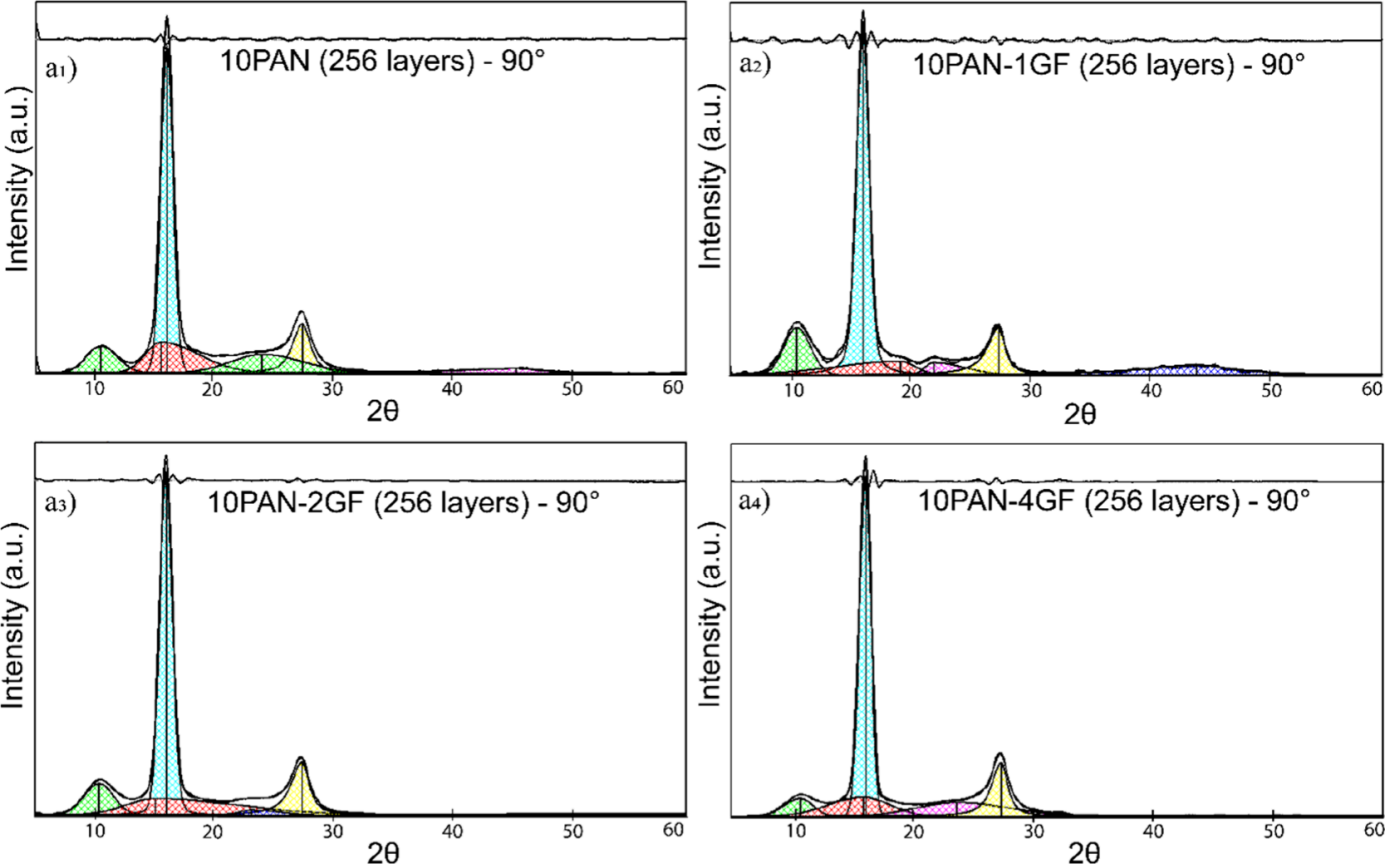
XRD pattern of PAN showing the separation of crystalline and amorphous regions to evaluate the relative degree of crystallinity. Crystallinity was determined by integrating the areas under the resolved peaks after subtracting a linear baseline established from 2θ = 5° to 60°. (a_1_–a_4_) XRD results for 256-layered fibers oriented at 90°.

To quantify crystallinity, peak deconvolution was performed using Jade software. A linear baseline was established between 2θ = 5° and 60°, and the area beneath this baseline was subtracted. The resulting diffraction patterns were then decomposed into crystalline and amorphous contributions to isolate the degree of crystallinity and calculate other crystal structure parameters, including crystal size (L), plane spacing (d), and peak sharpness (FWHM) (Tables 2 and 3), Bragg’s law (eq 2) and the Scherrer equation (eq 3) were employed to compute these values. The 17° peak represents folded-chain crystals, and the 29.5° peak further confirms the lamellar stacking typical of well-ordered PAN microstructures.

**Table 2:** Crystal Parameters of the (110) and (020) Planes for Various 256-Layered PAN Fiber Types—0°

Fiber type	High DR	Crystallinity (%)	2θ	Crystal plane (h,k,l)	Plane spacing d (nm)	Crystal size D (nm)	Ratio d (17°)/d (29°)	FWHM
10PAN	42.31	46.33	17.12	(110)	5.17	5.80	1.72	1.38
			29.78	(020)	3.00	3.94		2.04
10PAN-1GF	35.28	53.93	17.45	(110)	5.07	5.43	1.72	1.48
			30.22	(020)	2.95	3.61		2.28
10PAN-2GF	39.00	69.20	17.01	(110)	5.20	5.65	1.71	1.42
			29.35	(020)	3.04	4.42		2.79
10PAN-4GF	34.89	68.56	17.23	(110)	5.14	6.23	1.71	1.29
			29.66	(020)	3.00	4.78		1.71

**Table 3:** Crystal Parameters of the (110) and (020) Planes for Various 256-Layered PAN Fiber Types—90°

Fiber type	High DR	Crystallinity (%)	2θ	Crystal plane (h,k,l)	Plane spacing d (nm)	Crystal size D (nm)	Ratio d (17°)/d (29°)	FWHM
10PAN	42.31	54.80	17.09	(110)	5.18	6.00	1.72	1.34
			29.62	(020)	3.01	4.22		1.95
10PAN-1GF	35.28	60.58	17.28	(110)	5.13	6.00	1.71	1.34
			29.80	(020)	3.00	4.33		1.90
10PAN-2GF	39.00	63.59	17.07	(110)	5.20	5.90	1.72	1.36
			29.61	(020)	3.01	4.10		2.00
10PAN-4GF	34.89	62.92	17.40	(110)	5.09	6.43	1.70	1.25
			29.90	(020)	2.98	4.68		1.75

With increasing GF content, a significant improvement in crystalline features was observed. The diffraction peak at 2θ ≈ 17° became noticeably sharper and more intense with higher GF loading, particularly in the 10PAN-4GF sample, indicating enhanced molecular ordering. Crystallinity increased from 46.33% in pure PAN fibers to 69.20% in 10PAN-2GF and 68.56% in 10PAN-4GF fibers, nearly 45% enhancement. This crystallization behavior was accompanied by a subtle but measurable increase in the lateral crystallite size, from 5.80 nm (10PAN) to 6.23 nm (10PAN-4GF) for the (110) plane. Similarly, the (020) crystallite size increased from 3.94 to 4.78 nm (Table 2). These trends suggest that the introduction of GFs into the PAN matrix acts as a nucleating agent, improving chain alignment and promoting more efficient crystalline packing.^43^

Interestingly, the intermediate GF concentrations in the 10PAN-1GF and 10PAN-2GF samples exhibited lower crystallite sizes (5.43 and 5.65 nm, respectively), which may be due to the formation of voids or agglomerates. These structural imperfections may inhibit uniform chain packing, thereby reducing the apparent crystallite dimensions despite an overall increase in crystallinity.^44^ This observation also suggests the importance of optimal filler dispersion and loading thresholds to achieve balanced reinforcement and crystalline development.

Orientation-dependent scans further emphasized the influence of fiber processing and GF integration. At 0° (Figure 3a_1_–3a_4_), sharper peaks and higher intensity were consistently observed across samples compared to the 90° direction (Figure 4a_1_–4a_4_), which showed more diffused profiles. This contrast is indicative of anisotropic orientation along the fiber axis, particularly in high-draw ratio samples. The Herman’s orientation parameter, inferred from peak broadening and angular intensity distribution, suggested that GF inclusion facilitated better chain alignment and orientation along the spinning direction.^45^ This effect was most pronounced in 10PAN-4GF samples, supporting the hypothesis that well-dispersed GFs guide molecular alignment during draw-induced crystallization.^46^ Overall, the XRD data provides compelling evidence aligned with our hypothesis that turbine-derived GFs, when selectively dispersed, can significantly enhance the crystalline structure of PAN precursor fibers.^47^ The combination of increased crystallinity, expanded crystal size, and improved orientation contributes to the superior mechanical performance observed in PAN-GF composite fibers. These findings not only confirm the efficacy of glass fiber as a reinforcement additive but also highlight the potential of using layered fiber spinning and dry-jet wet spinning processes to tailor microstructures for high-performance carbon fiber precursors.

### 2.3 Mechanical Performance of Fibers

#### 2.3.1 Influence of GF Concentration

The tensile properties of the final drawn stage fibers in varying GF content and layer architectures are summarized in Table 4 and visualized in Figure 5. The incorporation of GF derived from WTB waste serves as an effective reinforcing strategy, significantly enhancing both stiffness and tensile strength of the precursor PAN-based composite fibers. For example, in the 32-layered group (Figure 5a_1_), the addition of 4 wt.% GF to the PAN matrix led to an increase in tensile strength from 301.72 ± 34.47 MPa (10PAN) to 347.85 ± 27.93 MPa (10PAN-4GF), while the Young’s modulus rose from 8.38 ± 0.07 GPa to 13.10 ± 0.60 GPa. This 56% increase in stiffness highlights the reinforcing capability of GF, likely due to their high aspect ratio, stiffness, and ability to constrain the PAN chains and transfer stress efficiently within the matrix.

**Figure 5:**
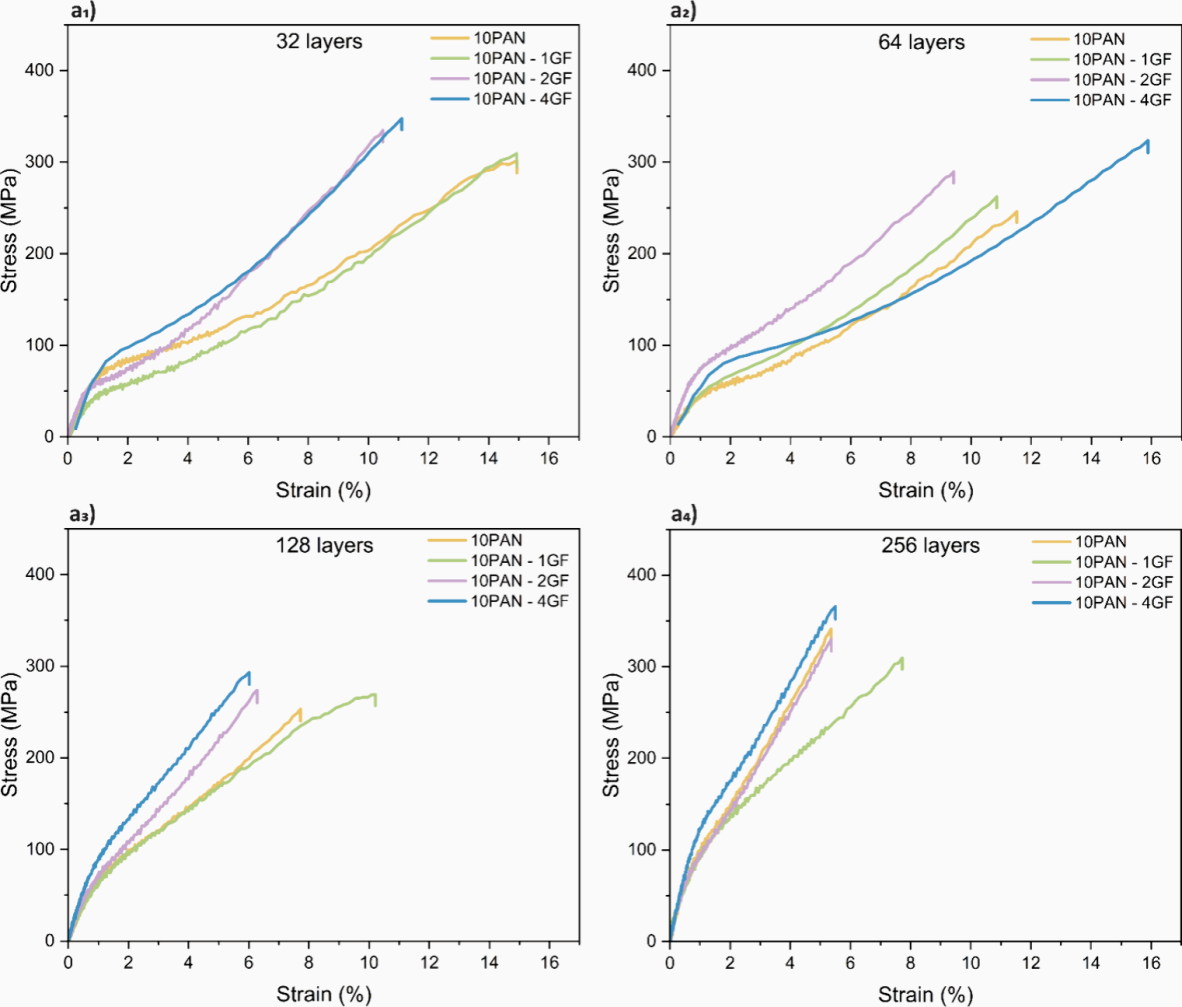
Tensile stress–strain behavior of precursor layered PAN and PAN-GF fibers with different compositions and number of layers: (a_1_) 32 layers, (a_2_) 64 layers, (a_3_) 128 layers, and (a_4_) 256 layers.

**Table 4:** Mechanical Properties of the Precursor Layered Fibers and Their Composition with the Layer Numbers

		Mechanical properties of prestabilized fibers			
Layers	Fiber type	Youngs Modulus (GPa)	Tensile Strength (MPa)	Elongation at break (%)	Diameter of maximum stretched fibers (μm)
32	10PAN	8.38 ± 0.07	301.72 ± 34.47	14.93 ± 2.92	70.10 ± 4.93
	10PAN-1GF	10.40 ± 0.23	309.38 ± 29.47	14.93 ± 4.45	87.23 ± 3.78
	10PAN-2GF	9.27 ± 0.56	334.86 ± 14.25	10.48 ± 2.60	72.14 ± 2.79
	10PAN-4GF	13.10 ± 0.60	347.85 ± 27.93	11.10 ± 4.31	69.95 ± 4.32
64	10PAN	6.95 ± 0.58	246.16 ± 15.05	11.52 ± 3.87	77.09 ± 2.16
	10PAN-1GF	8.61 ± 0.79	262.16 ± 15.05	10.85 ± 1.17	73.43 ± 4.11
	10PAN-2GF	10.36 ± 1.10	289.66 ± 29.93	9.42 ± 3.03	80.58 ± 1.29
	10PAN-4GF	7.14 ± 1.87	323.76 ± 34.52	16.13 ± 3.47	71.51 ± 2.02
128	10PAN	8.53 ± 0.02	253.76 ± 17.99	7.72 ± 1.15	77.25 ± 2.80
	10PAN-1GF	8.92 ± 0.31	266.65 ± 35.46	10.48 ± 1.29	75.44 ± 3.49
	10PAN-2GF	8.80 ± 0.69	273.83 ± 31.07	6.93 ± 0.34	74.28 ± 3.95
	10PAN-4GF	11.19 ± 1.28	293.20 ± 22.12	6.01 ± 0.89	75.07 ± 1.82
256	10PAN	12.86 ± 0.38	341.02 ± 22.74	5.36 ± 0.44	77.93 ± 2.04
	10PAN-1GF	10.02 ± 0.31	309.85 ± 12.32	7.72 ± 2.07	79.35 ± 2.71
	10PAN-2GF	12.78 ± 1.88	329.96 ± 24.32	5.26 ± 0.63	66.08 ± 2.53
	10PAN-4GF	14.49 ± 0.11	365.83 ± 19.51	5.50 ± 0.82	70.75 ± 2.99

As the GF concentration increases, improvements in mechanical properties are also seen in other layered configurations. In 64-layered composites (Figure 5a_2_), 10PAN-2GF achieved a modulus of 10.36 ± 1.10 GPa, representing a ∼49% increase over the corresponding pure PAN fiber (6.95 ± 0.58 GPa). Similarly, tensile strength increased from 246.16 ± 15.05 MPa (10PAN) to 289.66 ± 29.93 MPa (10PAN-2GF). However, the addition of 4 wt.% GF in this case resulted in 7.14 ± 1.87 GPa modulus and 323.76 ± 34.52 MPa tensile strength, indicating that while tensile strength improved, stiffness dropped relative to the 2 wt.% sample. This suggests potential issues related to dispersion or agglomeration of GF, which can act as stress concentrators or create internal voids that compromise structural integrity. In the 256-layered fibers (Figure 5a_4_), a similar trend is observed. 10PAN-4GF achieved the highest modulus of 14.49 ± 0.11 GPa and tensile strength of 365.83 ± 19.51 MPa, reflecting improved chain alignment and synergy between the filler and the PAN matrix. In contrast, 10PAN-1GF and 10PAN-2GF composites showed reduced moduli (10.02 ± 0.31 GPa and 12.78 ± 1.88 GPa, respectively), suggesting that optimal mechanical performance is not solely dictated by GF content but also by uniform distribution and interface bonding quality.

Noteworthy, a trade-off is evident between stiffness/strength and ductility, which is consistent with most literature reports.^48^ Elongation at break decreased with increasing GF concentration. For instance, in 32-layered fibers, the elongation dropped from 14.93 ± 2.92% (10PAN) to 10.48 ± 2.60% (10PAN-2GF), indicating reduced deformability due to the restricted chain mobility imposed by rigid GF particles. Therefore, to maintain a balance between mechanical reinforcement and toughness, optimizing GF content is critical for our future studies. Excessive filler loading beyond the percolation threshold may hinder PAN crystallinity or cause premature failure due to filler agglomerates or interfacial voids.

#### 2.3.2 The Fiber Layering Effects

The fiber layering strategy achieved through the multiplier stack in the dry-jet wet spinning setup plays a significant role in governing mechanical performance. As the number of layers increases from 32 to 256, the fibers exhibit improved mechanical properties, attributed to enhanced confinement effects and molecular alignment during drawing. This trend is clear in the stress–strain plots (Figure 5) and is substantiated by the mechanical data in Table 4. For example, in pure PAN fibers, increasing the number of layers from 32 to 256 resulted in an increase in modulus from 8.38 ± 0.07 GPa to 12.86 ± 0.38 GPa, and tensile strength from 301.72 ± 34.47 MPa to 341.02 ± 22.74 MPa. This improvement is largely attributed to the finer lamellae and higher draw-induced orientation (Figures 3 and 4, XRD data) facilitated by increased layering, which minimizes the diameter of each sublayer and reduces structural defects.

The effect of layering is also synergistic with GF reinforcement. For instance, 10PAN-4GF fibers showed a consistent increase in modulus with more layers from 13.10 ± 0.60 GPa (32 layers) to 14.49 ± 0.11 GPa (256 layers). Similarly, tensile strength improved from 347.85 ± 27.93 MPa to 365.83 ± 19.51 MPa (Figure S3). These trends validate the hypothesis that thinner individual layers in highly layered fibers promote better filler alignment, reduce defect propagation, and maximize filler–matrix interfacial area, all contributing to superior mechanical integrity. However, an important observation is the slight dip in performance for intermediate layer counts (e.g., 64 layers), particularly in the 10PAN-4GF case, where the modulus drops to 7.14 ± 1.87 GPa, likely due to nonuniform dispersion or ineffective stress transfer caused by processing-induced defects or filler agglomeration. This points to the importance of optimized flow conditions and shear alignment during spinning to ensure consistent layer structure and effective GF distribution.

Another significant consideration is the trade-off between strength and extensibility as layering increases. The elongation at break steadily decreases from 14.93% (10PAN, 32 layers) to 5.36% (10PAN, 256 layers), which reflects higher stiffness but reduced ductility due to constrained chain extension. For composites, this effect is even more pronounced due to the rigid inclusions. For example, 10PAN-4GF in 256-layer fibers shows an elongation of only 5.50 ± 0.82%, reinforcing the need for a balance between mechanical reinforcement and flexibility, especially in applications requiring both high load-bearing capacity and impact tolerance. From these mechanical analyses, it can be concluded that increasing the number of layers in PAN-GF composite fibers significantly enhances modulus and tensile strength, particularly at optimal GF loadings. However, care must be taken to manage dispersion, especially when the concentration is going beyond specific content, interfacial adhesion, and processing parameters, to avoid performance degradation due to microstructural inhomogeneities. The combination of layering and upcycled GF content from wind turbine blades provides a scalable, sustainable pathway for fabricating high-performance composite fibers.

Although the 256-layered PAN-GF composite fibers demonstrated the highest mechanical performance in this study, increasing the number of layers beyond this point does not necessarily lead to continued improvements. Initially, increasing the number of layers enhances mechanical properties such as tensile strength, Young’s modulus, and toughness by improving stress transfer between the polymer and GF. Thinner individual layers allow for better interfacial interactions and energy dissipation during fracture, such as 512-layered fibers. However, this trend only holds up to an optimal point. At very high layer counts, such as in 1024 and 2048-layered fibers, the layers become extremely thin, making it difficult to maintain their structural integrity, such as voids, microcracks, and poorly defined interfaces, all of which negatively impact mechanical performance.^29,41^ As a result, the fibers become more prone to premature failure and lose the mechanical advantages that multilayer structuring is intended to provide. Additionally, very thin layers can hinder proper GF dispersion, leading to aggregation and increased GF-to-GF contact, which reduces load transfer efficiency.

### 2.4 Dynamic Mechanical Analysis (DMA) of Fibers

DMA was conducted to study how variations in GF concentration and fiber layer number (Figure 6) affect the viscoelastic behavior and interfacial dynamics of the multilayered composite fibers. The evolution of the damping factor, tan­(δ), the ratio of the loss modulus to the storage modulus, provides information on the energy dissipation capacity of the material and the quality of the interfacial bonding between the PAN matrix and the GF fillers embedded.

**Figure 6:**
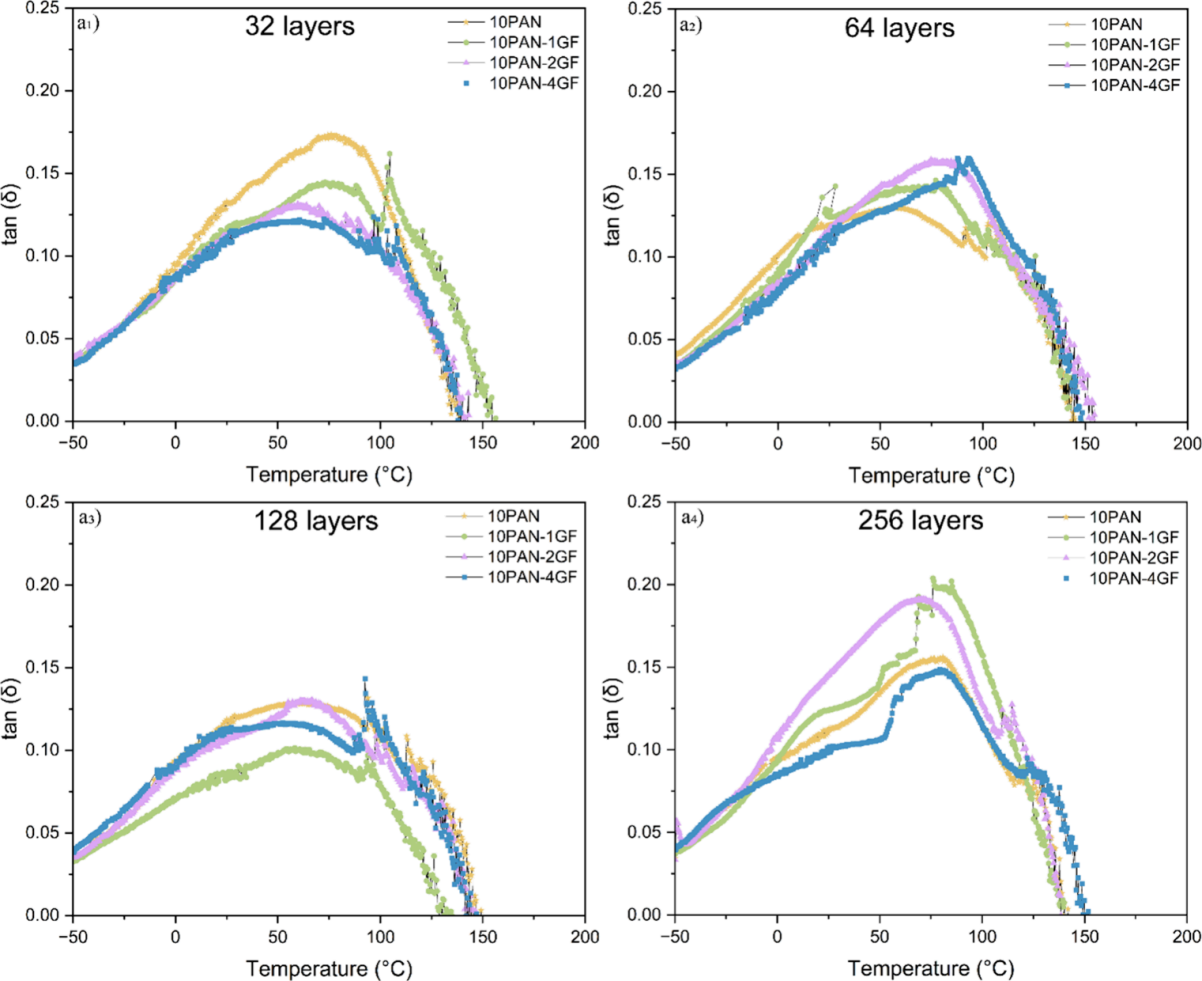
Dynamic mechanical analysis (DMA) of the fibers as a function of glass fiber concentration and layer number was conducted over a temperature range of −50 to 160 °C to evaluate the damping factor, tan­(δ) for (a_1_) 32 layers, (a_2_) 64 layers, (a_3_) 128 layers, (a_4_) 256 layers, defined as the ratio of the loss modulus to the storage modulus that provides insight into the material’s viscoelastic behavior.

During the temperature sweep, all fibers were subjected to a constant axial force of 1 N and eventually fractured due to thermal softening, as indicated by storage modulus profiles (Figure S4 and Table S4). Notably, the 256-layered fibers showed the highest failure temperature, which is indicative of superior thermal stability and mechanical resilience. This is consistent with the enhanced tensile performance observed in Table 4, where 10PAN-4GF exhibits the highest modulus (14.49 ± 0.11 GPa) and tensile strength (365.83 ± 19.51 MPa).

At room temperature, the initial tan­(δ) values progressively decrease with increasing fiber layer number, with the 128-layered fibers displaying the lowest tan­(δ), particularly in the 10PAN-4GF sample. This suppression of tan­(δ) reflects improved stiffness and elasticity, likely caused by restricted polymer chain mobility due to increased GF loading and enhanced interfacial bonding. In contrast, the pure PAN fibers consistently exhibit the highest tan­(δ) values at room temperature across all layering conditions, indicating a greater degree of polymer chain motion and lower filler-induced constraint. As temperature increases beyond the glass transition point (∼70 °C), the viscoelastic damping behavior becomes more distinct. For instance, in the 256-layered fibers (Figure 6a_4_), 10PAN-2GF exhibits the highest tan­(δ) peak (∼0.20), which suggests elevated interfacial energy dissipation. This can be attributed to imperfect GF dispersion and increased polymer chain mobility near the filler–matrix interface. Conversely, 10PAN-4GF shows the lowest damping response (∼0.15), highlighting superior interfacial adhesion and effective stress transfer, endorsed by its mechanical superiority seen in the tensile data.

It is worth mentioning that, for some intermediate-layered fibers (e.g., 64- and 128-layered, Figure 6a_2_ and Figure 6a_3_), GF inclusion results in nonmonotonic trends. For example, 10PAN-1GF in the 128-layer configuration displays the lowest tan­(δ), suggesting strong elastic recovery and reduced internal friction, attributes aligned with its relatively high tensile modulus (8.92 ± 0.31 GPa) and moderate tensile strength. These trends suggest that specific layer-to-filler ratios optimize interfacial coupling, while others may suffer from local agglomeration or void formation that introduce mechanical inconsistencies. The dynamic properties were summarized in SI (i.e., Figure S4 and Table S4), the tan­(δ) behavior across varying layer counts and GF concentrations aligns well with the static mechanical properties from tensile testing (Figure 5 and Table 4). Lower tan­(δ) values reflect better filler–matrix interaction and superior load transfer, resulting in higher tensile strength and modulus. Conversely, higher tan­(δ) values suggest increased damping, likely from interfacial slippage or polymer chain relaxation around poorly dispersed GFs. Thus, these DMA findings further validate the critical role of precise filler loading and structural control in achieving high-performance PAN-based composite fibers derived from wind turbine waste.

## 3 Conclusion

In summary, the comprehensive analysis of DSC, XRD, tensile, and dynamic mechanical data confirms that the incorporation of GFs derived from wind turbine waste significantly enhances the structural and mechanical performance of multilayered PAN composite fibers. As the number of layers increases in the PAN-GF composites, the individual layer thickness decreases, resulting in relatively thicker layers in 32-layered fibers and much thinner layers in the 256-layered ones. The presence of GFs introduces a templating effect that promotes higher crystallinity and larger crystallite sizes within the PAN matrix. This structural refinement strongly correlates with the observed improvements in tensile strength and modulus.

DSC revealed enhanced thermal stabilization, indicated by improved cyclization and oxidation behavior in GF-reinforced fibers. Specifically, for 256-layered fibers, the activation energy for cyclization increased by 17.75%, from 114.36 kJ/mol in 10PAN to 134.56 kJ/mol in 10PAN-1GF, demonstrating more efficient thermal stabilization. XRD analysis supported these findings, showing an increase in both crystallinity and crystal size with GF incorporation. For example, crystallinity rose significantly from 46.33% in 10PAN to 68.56% in 10PAN-4GF, confirming the role of GFs as effective nucleating agents. Tensile testing demonstrated marked improvements in mechanical properties with optimal GF content and layering architecture. In the case of 256-layered 10PAN-4GF fibers, the modulus increased by 16%, from 12.86 to 14.49 GPa, while the tensile strength improved by 7%, from 341.02 to 365.83 MPa. DMA further corroborated these enhancements, showing improved interfacial interactions and reduced damping behavior in high-layer, high-GF composites. For instance, among the 256-layered fibers, 10PAN-2GF exhibited the highest tan­(δ) peak (∼0.20), suggesting elevated interfacial energy dissipation. In contrast, 10PAN-4GF showed the lowest damping response (∼0.15), indicating stronger interfacial adhesion and more effective stress transfer, consistent with the tensile data. Together, these findings validate the potential of recycled GF as sustainable, high-performance reinforcements for advanced structural fiber applications.

## 4 Experimental Section

### 4.1 Materials

The PAN copolymer used in this study consisted of 99.5% acrylonitrile and 0.5% methacrylate, with a molecular weight of 230,000 g/mol and an average particle size of 50 μm, sourced from Goodfellow Cambridge Limited, Huntingdon, England. The solid waste from the WTB was obtained from TPI Composites, Inc., Iowa, USA. The WTBs, composed of wood, adhesives, coatings, and GF, were processed by breaking down the composite material and reducing particle size through sequential steps, including shredding, crushing, milling, grinding, and sieving (using a 40-mesh screen) as seen in Figure S5. This process yielded fine particles with a glass fiber concentration of approximately 82 wt.%, along with residual adhesives, wood, coatings, and thermoset fragments. ImageJ software was used to estimate the average particle size of processed GFRP, approximately 40 μm in length and 4 μm in diameter. The solvents used included N, N-dimethylformamide (DMF) (ACS reagent, ≥99.8%) to dissolve PAN and disperse GF, and methanol (ACS reagent, ≥99.8%) as a coagulant, both obtained from Sigma-Aldrich, USA. All materials were purchased and used as received, without further modification.

### 4.2 PAN/GF Layered Fibers Preparation

The following section describes the spinning of multilayered fibers.

Preparation of multilayered PAN and PAN-GF feedstocks: 8 g of PAN was dissolved in 80 mL of DMF to create a spinning batch. Initially, GF was first incorporated at concentrations of 1–4 wt.% w.r.t PAN in the DMF solvent, resulting in a suspension achieved by tip sonication for 30 min at an amplitude of 60% (Q500, Fisher Scientific, US). Subsequently, PAN was added to the solvent to create the PAN-GF composite solution for feedstock B, while the pure PAN solution was used as feedstock A for parallel layers in spun fibers. Both mixtures were stirred mechanically at 130 °C for 45 min until a clear solution was achieved. To eliminate air bubbles, the solutions were placed in a vacuum oven (Lindberg Blue M lab oven, Thermo Scientific US) at 50 °C for 30 min. Following deaeration, the solutions were transferred to a metal syringe connected to a pump for fiber spinning, which were injected into a multilayered spinneret at a controlled rate of 2 mL/min to facilitate the extrusion and formation of fibers. This unique multilayered spinneret was manufactured using Inconel alloys on a Concept Laser 2 metal 3D printer, allowing for intricate designs and precise control of the fiber spinneret.

#### 4.2.1 Dry-Jet Wet Spinning of Multilayered PAN and PAN-GF Fibers

##### 4.2.1.1 Fiber Spinning

The solution was injected into an air gap of 1.5–2.0 cm before entering the coagulation bath. In the dry-jet wet spinning, the air gap facilitated fiber extension, reducing defect density and promoting molecular alignment. Immersion in the coagulation bath triggered two simultaneous diffusion processes: the polymer-rich phase condensed into the fiber, while the solvent-rich phase (i.e., DMF) exchanged with the nonsolvent (i.e., methanol), forming a gel-like fiber structure. The as-spun fibers were then soaked in methanol for 30 min for coagulation. The coagulation rate needed to be high enough to minimize gradient differences between the surface and core, ensuring a uniform coagulation procedure and preventing core deformation, maintaining a circular fiber cross-section. However, irregular cross-sectional shapes could develop if diffusion rates between layers were mismatched, causing a gradient in the polymer distribution. Flow and injection rates were critical in determining fiber dimensions and chain alignment. Higher flow rates through the coagulation bath and lower injection rates for the spinning solution resulted in a lower fiber diameter and higher defect density. However, a larger draw ratio during coagulation did not always guarantee better polymer chain alignment due to competing effects between polymer stretching and molecular recoiling. For example, excessive stretching from high flow rates could lead to molecular recoil, hindering optimal polymer chain alignment. Therefore, the injection rates were optimized at 2 mL/min for consistent fiber collection onto the winding apparatus.

##### 4.2.1.2 Fiber Drawing

During hot drawing, fibers were stretched through baths of water and silicone oil to their maximum draw ratios without breaking. High shear forces aligned the macromolecules parallel to the fiber axis. Initially, the fibers were drawn through a water bath at 85 °C to facilitate polymer chain alignment and remove DMF solvent. They were then soaked in methanol for 24 h to enhance coagulation and minimize the DMF content in the gel fibers. The wet PAN fibers were dried at 50 °C under vacuum for 30 min to remove moisture and collapse voids. The fibers were then drawn in an oil bath at 125, 135, and 145 °C to produce the highest draw ratio fibers at 145 °C (Tables 5 and S5). This high-temperature drawing maximized molecular extension, and this newly formed surface applied a protective layer to the fibers. The increasing bath temperature helped to orient the GF by overcoming the rotational momentum of the GF. Stretching at higher temperatures also enhanced PAN fiber molecular orientation and created dense structures with improved mechanical properties.

**Table 5:** Summary of Layers, Compositions, Fiber Type, Drawing Parameters, and Layer Confinement

		Composition wt %		Drawing results			
Layers	Fiber type	Feedstock A (PAN wt %)	Feedstock B (GF wt % w.r.t PAN)	Processing of fibers (fiber-drawing)	High Draw ratio (DR6)	Diameter of manually stretched fiber (μm)	Individual layer width (μm)
32	10PAN	10	0	Precursor fibers were drawn in water at 85 °C and in silicone oil at 125 °C, 135 °C, and 145 °C to produce high DR fibers.	38.76	70.10 ± 4.93	2.19
	10PAN-1GF		1		49.37	87.23 ± 3.78	2.72
	10PAN-2GF		2		44.69	72.14 ± 2.79	2.25
	10PAN-4GF		4		57.68	69.95 ± 4.32	2.18
64	10PAN		0		41.68	77.09 ± 2.16	1.20
	10PAN-1GF		1		45.21	73.43 ± 4.11	1.14
	10PAN-2GF		2		45.63	80.58 ± 1.29	1.26
	10PAN-4GF		4		48.54	71.51 ± 2.02	1.12
128	10PAN		0		45.32	77.25 ± 2.80	0.60
	10PAN-1GF		1		45.14	75.44 ± 3.49	0.59
	10PAN-2GF		2		42.20	74.28 ± 3.95	0.58
	10PAN-4GF		4		48.74	75.07 ± 1.82	0.58
256	10PAN		0		42.31	77.93 ± 2.04	0.30
	10PAN-1GF		1		35.28	79.35 ± 2.71	0.31
	10PAN-2GF		2		39.00	66.08 ± 2.53	0.26
	10PAN-4GF		4		34.89	70.75 ± 2.99	0.28

### 4.3 Characterizations

DSC (DSC 250, TA Instruments Inc., USA) was performed on a single fiber sample (∼2 mg) for each fiber type. The temperature increased from room temperature (25 °C) to 350 °C with a heating rate ranging from 5 to 15 °C/min in a nitrogen atmosphere to understand the cyclization behaviors, followed by measurements in air to evaluate the oxidation and cross-linking behavior.

XRD patterns were collected using a Bruker D2 Phaser operating at 40 kV and 30 mA with Cu Kα radiation (λ = 0.1541 nm). The diffraction peaks observed at 2θ ≈ 17° and 29° were analyzed with Jade software to determine the degree of crystallinity and the interplanar spacing (d) using Bragg’s law (eq 2). The crystallite size (D) was subsequently calculated from these parameters using the Scherrer eq (eq 3). Each measurement was performed on bundles of 10 fibers of each type.

nλ=2d⁡sin⁡θ 2

$$D=\frac{K\lambda }{\beta \cos\theta }$$ 3

where *D* = crystallite size (in nm), *K* = shape factor (0.9), λ = X-ray wavelength (0.15406 nm for Cu Kα radiation), β = full width at half-maximum (FWHM) of the peak (in radians), θ = Bragg angle (half of the measured 2θ in radians).

Single fiber tensile testing was performed using a Discovery hybrid rheometer-2 (DHR-2, TA Instruments Inc., USA) with a gauge length of 20 mm and a constant linear strain rate of 50 μm/s, applied to the highest draw ratio fibers. For each fiber type, 8–10 individual fibers were tested to evaluate their mechanical properties, including Young’s modulus, tensile strength, and tensile strain at break.

DMA was performed using a DHR-2 rheometer in tension mode over a temperature range of −50 to 160 °C, with a heating rate of 3 °C/min. The frequency was maintained at 1 Hz throughout the test. To ensure purely elastic deformation of the fibers, the prestrain was limited to 0.25% for a 25 mm gauge length, with a minimum applied force of 1N and an oscillatory strain amplitude of 0.2%. During the temperature sweep, the storage modulus (E), loss modulus (E), and damping factor (tan δ) were continuously recorded. Each test was conducted on bundles comprising 10 fibers of each type.

## Supplementary Material



## Notes

Differential scanning calorimetry (DSC) performed under both nitrogen and air atmospheres to determine peak temperatures and enthalpy changes across different fiber layer configurations, including 256-layered fibers subjected to varying draw ratios; mechanical testing of PAN-GF fibers comparing the performance of 32-layered and 256-layered configurations using weights; dynamic mechanical analysis (DMA) of high draw ratio fibers with varying glass fiber concentrations and layer numbers revealing trends in storage modulus, loss modulus, and tan delta at room temperature, as well as corresponding glass transition temperatures; the mechanical recycling process of wind turbine blade (WTB) solid waste involving sequential shredding, crushing, milling, grinding, and sieving steps to achieve uniform particle sizes suitable for fiber production; fiber spinning rationale (PDF)
