# Supplementary material for: Turbine-to-Textile: Upcycling Wind Turbine Blade Waste into High-Performance PAN Composite Fibers
Source: ACS Appl Polym Mater. 2025 Oct 17;7(21):14188–200. doi: 10.1021/acsapm.5c02466 (PMC12624527; doi:10.1021/acsapm.5c02466)
Supplement: Supplementary file 1 [file ap5c02466_si_001.pdf]

Supporting Information

Turbine-to-Textile: Upcycling Wind Turbine Blade Waste into High-Performance PAN Composite Fibers

Varunkumar Thippanna[1], Xiao Sun[2], M. Taylor Sobczak[1], Arunachalam Ramanathan[1], Taylor G Theobald[1], Ian Doran[1], Joshua Were[1], Libin Yang[1], James Jaraczewski[1], James Casey[3], Vladislav V Klepov[3], Liang Liang[4], Stephen Nolet[5], Arunachala Nadar Mada Kannan[6], Xin Xu[7], Kenan Song[8]*

[1]Mechanical Engineering, College of Engineering, University of Georgia, 302 E Campus Rd, Athens, 30602, GA, United States.

[2]Department of Mechanical and Industrial Engineering, College of Engineering, Northeastern University, 360 Huntington Ave, Boston, 02115, MA, United States.

[3]Department of Chemistry, University of Georgia (UGA), 302 E. Campus Rd., Athens, 30602, GA, United States.

[4]123DTechs., Inc., Athens, Georgia 30602, United States

[5]TPI Composites, Scottsdale, Arizona 85253, United States.

[6]Professor, The Polytechnic School (TPS), Ira Fulton Schools of Engineering, Arizona State University, Mesa, AZ, 85212, USA.

[7]Assistant Professor, The Polytechnic School (TPS), Ira Fulton Schools of Engineering, Arizona State University, Mesa, AZ, 85212, USA.

[8*]Associate Professor, Mechanical Engineering, School of Environmental, Civil, Agricultural and Mechanical Engineering (ECAM) & School of Chemical, Materials and Biomedical Engineering, College of Engineering, University of Georgia (UGA), 302 E. Campus Rd., Athens, 30602, GA, United States.

*Corresponding author, Email: kenan.song@uga.edu

Number of Pages: 13

Number of Figures: 5

Number of Tables: 5

Table of Contents

1. DSC – Kinetic analysis of fibers 5

2. Mechanical analysis of fibers 8

3. Dynamic Mechanical Analysis (DMA) of fibers 9

4. Mechanical recycling of Wind Turbine Blades 11

5. Fiber spinning rationale 12

6. References 13

Table of Figures

Figure S1. DSC curves of fibers in a nitrogen atmosphere at a constant heating rate of 10 °C/min for (a_1) 10PAN fibers, (a_2) 10PAN-1GF fibers, (a_3) 10PAN-2GF fibers, and (a_4) 10PAN-4GF fibers, illustrating the thermal behavior and peak temperatures. 6

Figure S2. DSC curves of 256-layered 10PAN and 10PAN-1GF fibers at various draw ratios, measured in a nitrogen (N_2) atmosphere at a constant heating rate of 10 °C/min, highlighting the thermal transitions influenced by fiber draw ratio. 7

Figure S3. Mechanical analysis of 10PAN-1GF fibers comparing 32-layered and 256-layered configurations, using weights 8

Figure S4. DMA analysis of high draw ratio fibers illustrating the effects of varying filler concentrations and layer numbers on storage modulus and loss modulus. 10

Figure S5. Mechanical recycling of wind turbine solid waste for PAN-based fiber production involves sequential processes such as shredding, crushing, milling, grinding, and sieving to obtain uniform particle size. 11

Table of Tables

Table S1. Peak temperature and enthalpy of 256-layered 10PAN and 10PAN-1GF fibers at varying heating rates in both nitrogen and air atmospheres to determine the activation energy via the Kissinger equation. 5

Table S2. Peak temperature and enthalpy values of different layered fiber types measured under a nitrogen (N_2) atmosphere 5

Table S3. Peak temperature and enthalpy of each draw ratio (DR) fiber for 256-layered 10PAN and 10PAN-1GF fibers, measured in a nitrogen (N_2) atmosphere at a constant heating rate of 10 °C/min........ 7

Table S4. DMA results of high draw ratio fibers with varying filler concentrations and layer number, showing storage modulus, loss modulus, tan delta at room temperature, and corresponding glass transition temperatures........ 9

Table S5. Table summarizing the fiber layer numbers, their compositions, processing, and drawing capabilities. 12

1. DSC – Kinetic analysis of fibers

Table S1. Peak temperature and enthalpy of 256-layered 10PAN and 10PAN-1GF fibers at varying heating rates in both nitrogen and air atmospheres to determine the activation energy via the Kissinger equation.

Heating rate (°C/min)	In N_2				In Air			
	10PAN DR6		10PAN-1GF DR6		10PAN DR6		10PAN-1GF DR6	
	T_{cyc} (°C)	Enthalpy (J/g)	T_{cyc} (°C)	Enthalpy (J/g)	T_{oxi} (°C)	Enthalpy (J/g)	T_{oxi} (°C)	Enthalpy (J/g)
5	287.45	447.78	292.45	332.82	291.18	380.47	291.90	685.29
7.5	298.40	362.90	299.55	196.40	298.01	330.65	298.98	445.49
10	303.68	325.59	304.62	301.05	302.99	298.55	304.37	343.09
12.5	308.41	300.76	309.10	243.63	306.73	257.26	308.99	335.06
15	312.87	291.67	313.16	217.20	312.15	1065.6	312.29	534.43

Table S2. Peak temperature and enthalpy values of different layered fiber types measured under a nitrogen (N_2) atmosphere

Layers	Fiber type (high DR)	Peak temperature T_{cyc} (°C)	Enthalpy (J/g)
32	10PAN	304.90	354.01
	10PAN-1GF	303.51	354.81
	10PAN-2GF	304.38	604.98
	10PAN-4GF	301.86	425.68
64	10PAN	304.31	140.91
	10PAN-1GF	306.96	504.17
	10PAN-2GF	305.60	293.73
	10PAN-4GF	305.62	267.49
128	10PAN	302.86	310.46
	10PAN-1GF	302.37	278.35
	10PAN-2GF	304.68	158.43
	10PAN-4GF	304.23	215.90
256	10PAN	303.68	325.59
	10PAN-1GF	304.62	293.58
	10PAN-2GF	302.47	288.51
	10PAN-4GF	305.17	379.56

The differential scanning calorimetry (DSC) analysis of multilayered PAN and PAN-GF fibers under nitrogen atmosphere reveals important insights into the thermal stabilization behavior with varying layer counts and glass fiber (GF) concentrations. As shown in **Figure S1** and **Table S1**, all fiber samples exhibit sharp exothermic peaks in the temperature range of ~300–307 °C, corresponding to the cyclization of PAN chains. Notably, 10PAN-1GF fibers with 64 layers show the highest peak temperature (306.96 °C), indicating a delayed onset of cyclization due to improved thermal stability from GF inclusion. This enhancement can be attributed to the interfacial interactions and improved thermal conductivity of GFs, which regulate heat transfer within the matrix.[1]

The enthalpy values also suggest that the inclusion of GFs and an increase in layer number substantially influence the energy absorbed during cyclization. For example, 10PAN-2GF with 32 layers exhibits a remarkably high enthalpy (604.98 J/g), signifying extensive molecular rearrangement and stabilization

facilitated by well-dispersed GFs. However, higher GF concentrations (such as 10PAN-4GF) may lead to filler agglomeration or void formation, which is consistent with our discussions in the manuscript, slightly reducing enthalpy and peak temperatures in some cases, as seen in the 128-layered fibers. These observations reinforce that moderate GF loading with proper dispersion and optimized layer confinement promotes more efficient thermal transitions and enhances the stability of the PAN-GF matrix. Overall, the DSC data underscores the synergistic effect of glass fiber concentration and multilayer structuring in tailoring the cyclization behavior of PAN-based precursor fibers for carbon fiber manufacturing.[2,3]

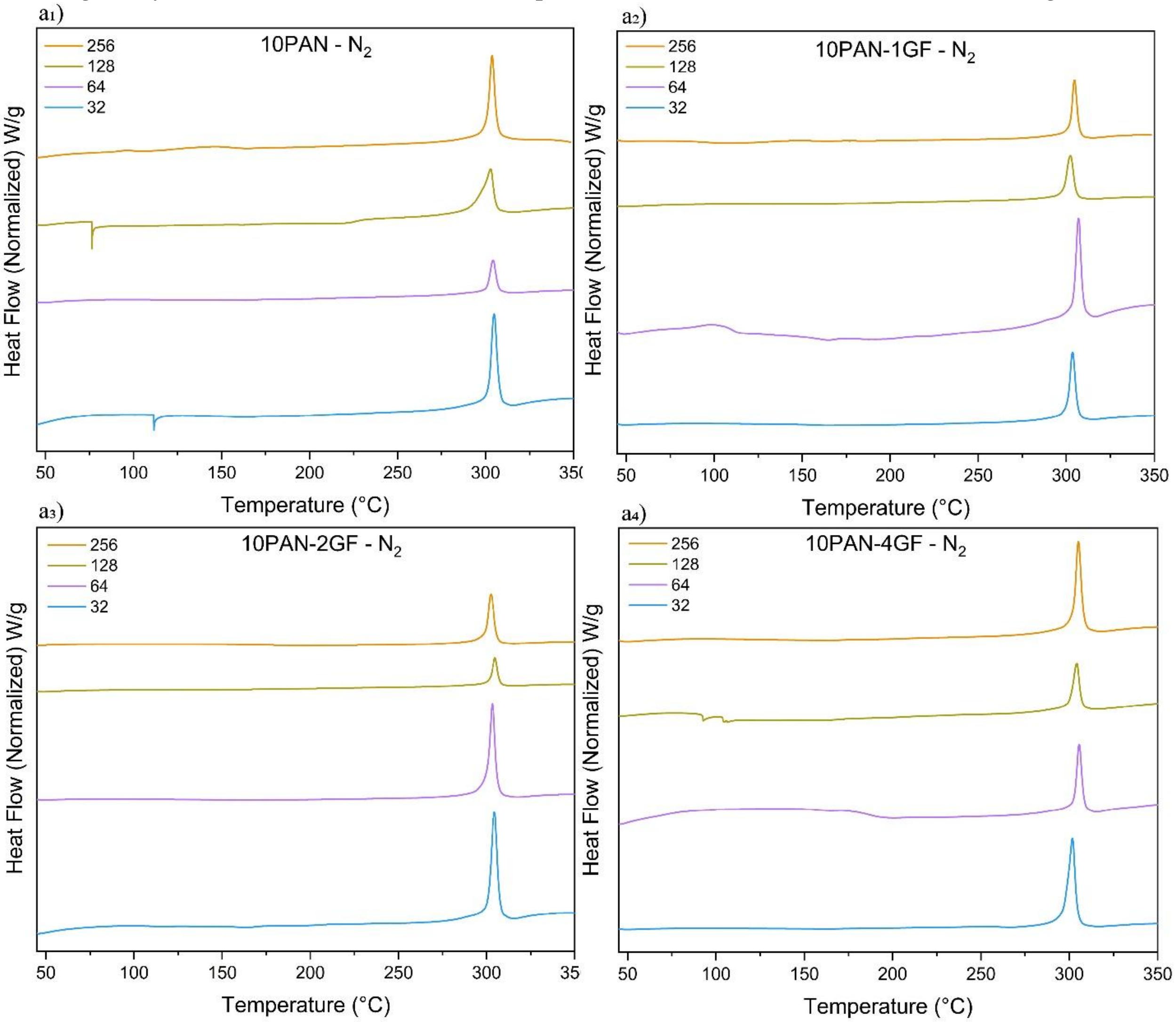

Figure S1. DSC curves of fibers in a nitrogen atmosphere at a constant heating rate of 10 °C/min for (a_1) 10PAN fibers, (a_2) 10PAN-1GF fibers, (a_3) 10PAN-2GF fibers, and (a_4) 10PAN-4GF fibers, illustrating the thermal behavior and peak temperatures.

Table S3. Peak temperature and enthalpy of each draw ratio (DR) fiber for 256-layered 10PAN and 10PAN-1GF fibers, measured in a nitrogen (N_2) atmosphere at a constant heating rate of 10 °C/min.

Fiber type	Draw Ratio (DR)	Peak Temperature T_{cyc} (°C)	Enthalpy (J/g)
10PAN	As Spun	281.48	456.64
	DR1	282.84	536.02
	DR2	287.16	598.55
	DR3	292.80	416.56
	DR4	298.65	204.80
	DR5	297.38	223.41
	DR6	303.68	325.59
10PAN-1GF	As Spun	274.23	606.07
	DR1	283.97	458.49
	DR2	290.24	514.14
	DR3	291.39	515.27
	DR4	298.04	257.75
	DR5	303.38	433.17
	DR6	303.54	1452.3

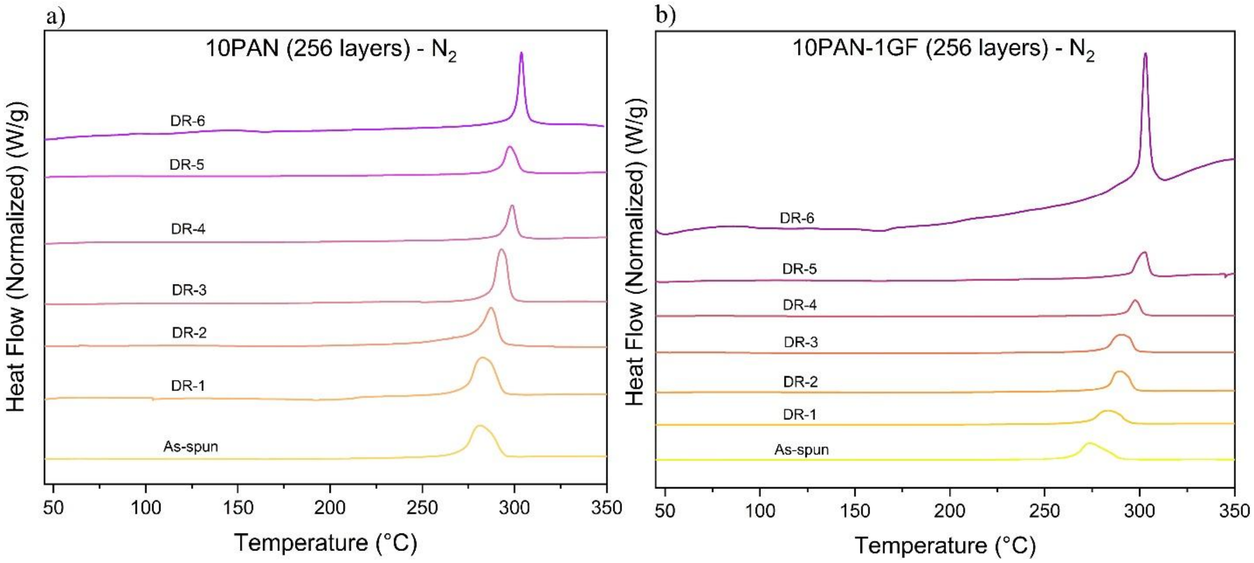

Figure S2. DSC curves of 256-layered 10PAN and 10PAN-1GF fibers at various draw ratios, measured in a nitrogen (N_2) atmosphere at a constant heating rate of 10 °C/min, highlighting the thermal transitions influenced by fiber draw ratio.

2. Mechanical analysis of fibers

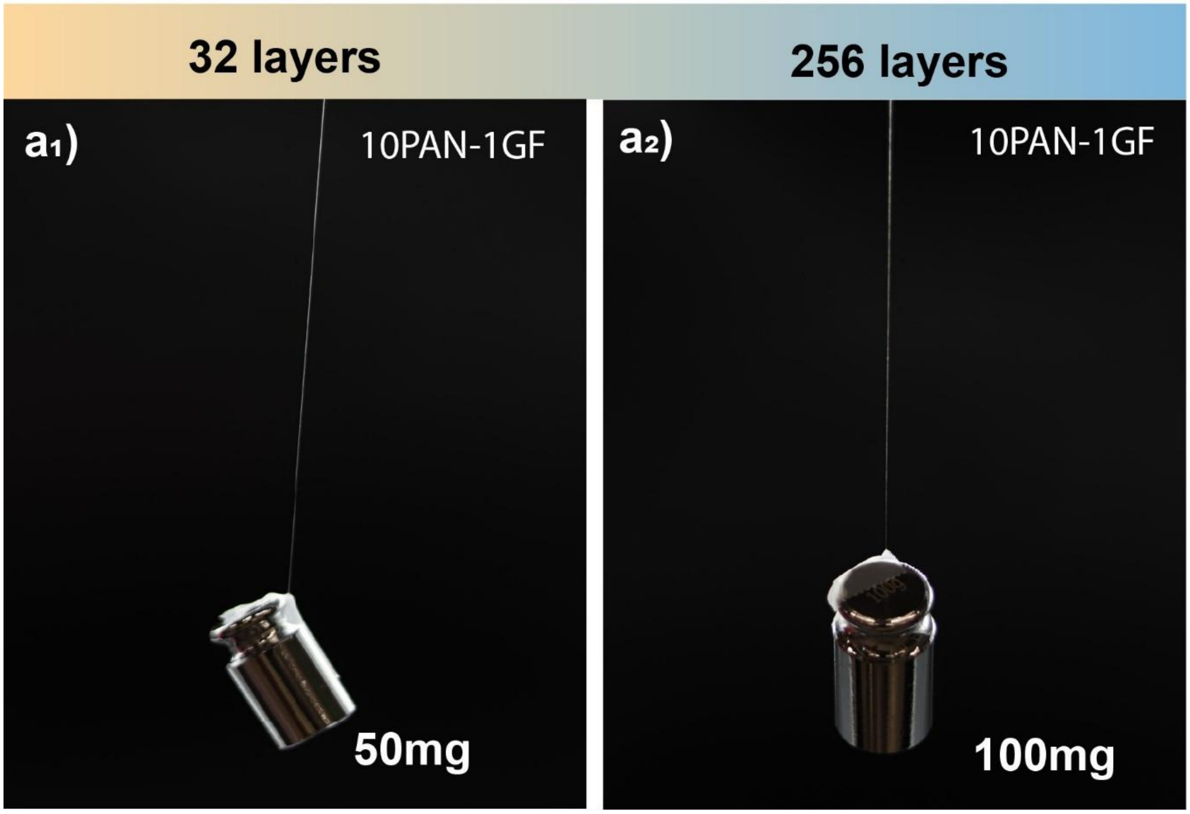

Figure S3. Mechanical analysis of 10PAN-1GF fibers comparing 32-layered and 256-layered configurations, using weights

3. Dynamic Mechanical Analysis (DMA) of fibers

The dynamic mechanical analysis (DMA) results summarized in **Table S4** provide key insights into the temperature-dependent mechanical behavior of multilayered PAN and PAN-GF composite fibers. At room temperature (25 °C), the storage modulus increases notably with the inclusion of glass fibers, particularly in the 10PAN-2GF and 10PAN-1GF samples, indicating improved stiffness due to the reinforcing effect of well-dispersed GFs. For instance, 10PAN-2GF at 32 and 256 layers exhibits the highest storage modulus of 16.31 GPa and 16.61 GPa, respectively, which correlates well with the enhanced crystallinity and crystal size observed in XRD results. Additionally, the reduced tan δ values for composite fibers (especially 10PAN-1GF and 10PAN-4GF at 128 layers) suggest better interfacial adhesion and restricted chain mobility, consistent with the stress transfer efficiency observed in tensile tests.[4] The glass transition temperatures (T_g) also shift depending on the fiber composition; higher T_g in some composite fibers (e.g., 10PAN-4GF at 64 and 256 layers) reflects enhanced thermal stability, in line with the DSC data.

Table S4. DMA results of high draw ratio fibers with varying filler concentrations and layer number, showing storage modulus, loss modulus, tan delta at room temperature, and corresponding glass transition temperatures.

Layers	Fiber type (DR6)	Temperature range (-50ºC to 150ºC)			
		Storage modulus (GPa) at RT (25ºC)	Loss modulus (GPa) at RT	Tan δ at T_g	Glass transition temp. (T_g)
32	10PAN	10.69	1.42	0.173	76.02
	10PAN-1GF	13.54	1.54	0.144	73.05
	10PAN-2GF	16.31	1.83	0.130	63.96
	10PAN-4GF	13.23	1.45	0.121	60.46
64	10PAN	9.50	1.13	0.130	55.32
	10PAN-1GF	13.30	1.66	0.144	77.24
	10PAN-2GF	6.91	0.81	0.158	74.74
	10PAN-4GF	13.25	1.45	0.148	82.27
128	10PAN	8.23	0.98	0.128	63.87
	10PAN-1GF	12.81	1.05	0.101	58.26
	10PAN-2GF	6.30	0.67	0.130	66.44
	10PAN-4GF	7.70	0.86	0.116	53.28
256	10PAN	6.06	0.68	0.156	80.86
	10PAN-1GF	13.01	1.61	0.203	75.95
	10PAN-2GF	16.61	2.41	0.191	71.37
	10PAN-4GF	10.11	1.03	0.148	79.36

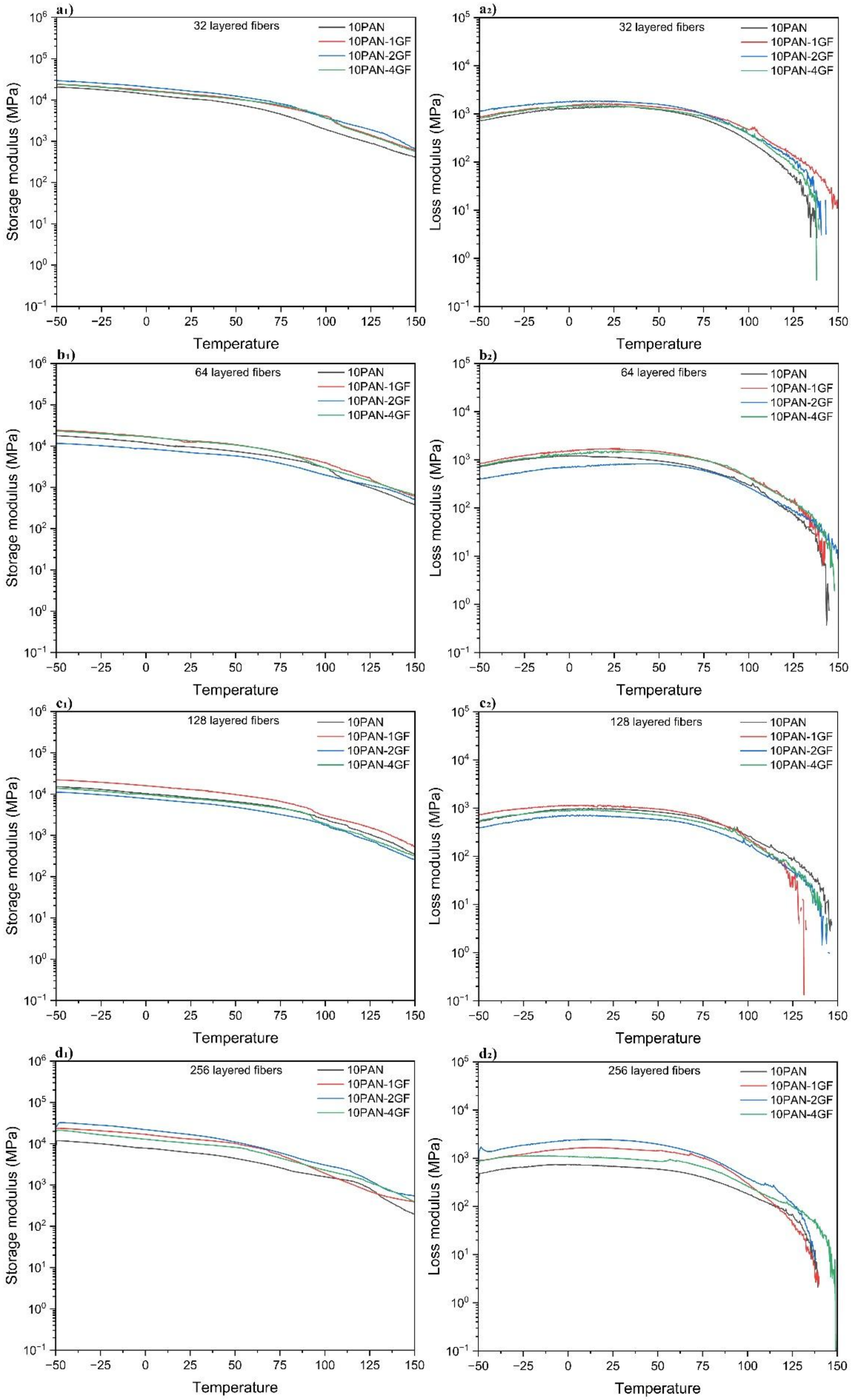

Figure S4. DMA analysis of high draw ratio fibers illustrating the effects of varying filler concentrations and layer numbers on storage modulus and loss modulus.

4. Mechanical recycling of Wind Turbine Blades

Figure S5 illustrates the stepwise mechanical recycling process of wind turbine blade (WTB) solid waste into fine particles suitable for PAN-based composite fiber fabrication. The process begins with shredding and crushing the bulk WTB waste into coarse fragments, followed by milling and grinding to reduce the particle size. The final sieving step ensures particle size uniformity, resulting in a graded series of powders from 2–3.5 mm down to approximately 38 microns. This scalable, solvent-free approach enables the incorporation of high-glass-fiber-content particles into polymer matrices, providing a viable route to upcycle wind turbine waste into value-added functional materials.[5,6]

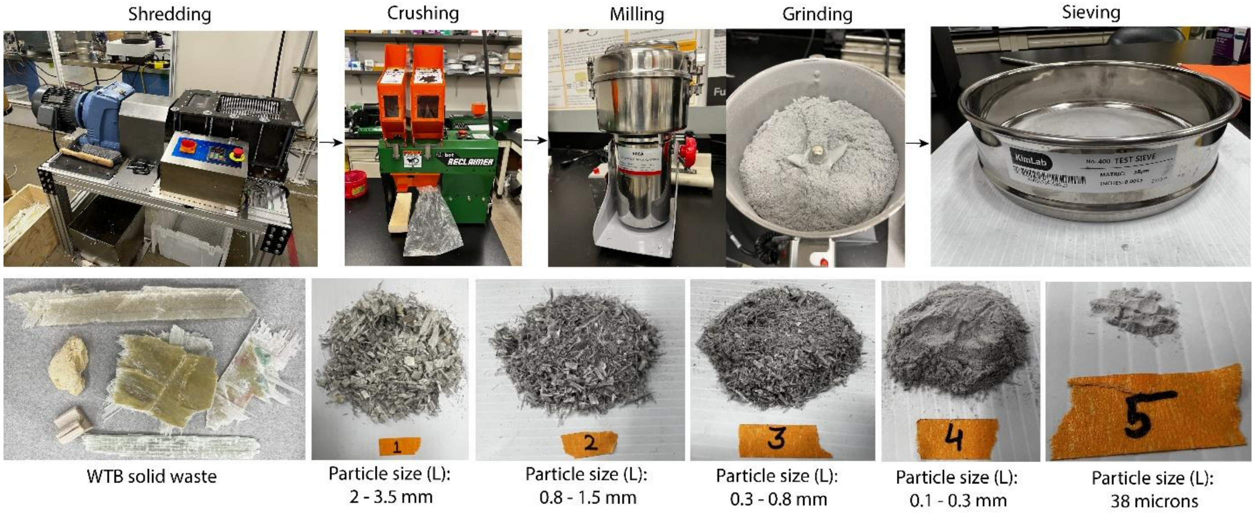

Figure S5. Mechanical recycling of wind turbine solid waste for PAN-based fiber production involves sequential processes such as shredding, crushing, milling, grinding, and sieving to obtain uniform particle size.

5. Fiber spinning rationale

Table S5. Table summarizing the fiber layer numbers, their compositions, processing, and drawing capabilities.

**Layers**	**Fiber type**	**Spinning medium**	**Draw ratio (DR) steps**	**Draw ratio**
32	10PAN	Oil (145 °C)	DR6	38.76
	10PAN-1GF			49.37
	10PAN-2GF			44.69
	10PAN-4GF			57.68
64	10PAN			41.68
	10PAN-1GF			45.20
	10PAN-2GF			45.63
	10PAN-4GF			48.54
128	10PAN			45.32
	10PAN-1GF			45.14
	10PAN-2GF			42.20
	10PAN-4GF			48.74
256	10PAN	Water (85 °C)	DR1	2.20
			DR2	5.68
			DR3	7.89
		Oil (125 °C)	DR4	14.91
		Oil (135 °C)	DR5	24.45
		Oil (145 °C)	DR6	42.31
	10PAN-1GF	Water (85 °C)	DR1	2.43
			DR2	3.91
			DR3	7.55
		Oil (125 °C)	DR4	13.21
		Oil (135 °C)	DR5	20.88
		Oil (145 °C)	DR6	35.28
	10PAN-2GF	Oil (145 °C)	DR6	39.00
	10PAN-4GF			34.89

6. References

(1) Lee, J. E.; Chae, Y. K.; Lee, D. J.; Choi, J.; Chae, H. G.; Kim, T. H.; Lee, S. Microstructural Evolution of Polyacrylonitrile Fibers during Industry-Mimicking Continuous Stabilization. *Carbon* **2022**, *195*, 165–173.

(2) Chang, H.; Lu, M.; Luo, J.; Park, J. G.; Liang, R.; Park, C.; Kumar, S. Polyacrylonitrile/Boron Nitride Nanotubes Composite Precursor and Carbon Fibers. *Carbon* **2019**, *147*, 419–426.

(3) Ramachandran, J.; Lu, M.; Arias-Monje, P. J.; Kirmani, M. H.; Shirolkar, N.; Kumar, S. Towards Designing Strong Porous Carbon Fibers through Gel Spinning of Polymer Blends. *Carbon* **2021**, *173*, 724–735.

(4) Xu, W.; Ravichandran, D.; Jambhulkar, S.; Zhu, Y.; Song, K. Hierarchically Structured Composite Fibers for Real Nanoscale Manipulation of Carbon Nanotubes. *Adv Funct Mater* **2021**, *31* (14), 2009311.

(5) Xu, M. xin; Ji, H. wen; Wu, Y. chang; Di, J. yi; Meng, X. xi; Jiang, H.; Lu, Q. The Pyrolysis of End-of-Life Wind Turbine Blades under Different Atmospheres and Their Effects on the Recovered Glass Fibers. *Compos B Eng* **2023**, *251*, 110493.

(6) Khalid, M. Y.; Arif, Z. U.; Hossain, M.; Umer, R. Recycling of Wind Turbine Blades through Modern Recycling Technologies: A Road to Zero Waste. *Renewable Energy Focus* **2023**, *44*, 373–389.
